# Differential Kv1.3, KCa3.1, and Kir2.1 expression in “classically” and “alternatively” activated microglia

**DOI:** 10.1002/glia.23078

**Published:** 2016-10-03

**Authors:** Hai M. Nguyen, Eva M. Grössinger, Makoto Horiuchi, Kyle W. Davis, Lee‐Way Jin, Izumi Maezawa, Heike Wulff

**Affiliations:** ^1^ Department of PharmacologyUniversity of CaliforniaDavisCalifornia; ^2^ Department of Pathology and Laboratory MedicineUniversity of California Davis Medical CenterSacramentoCalifornia; ^3^ M.I.N.D. Institute, University of California Davis Medical CenterDavis, SacramentoCalifornia

**Keywords:** microglia, potassium channel, Kv1.3, KCa3.1, Kir2.1, TRAM‐34, PAP‐1

## Abstract

Microglia are highly plastic cells that can assume different phenotypes in response to microenvironmental signals. Lipopolysaccharide (LPS) and interferon‐γ (IFN‐γ) promote differentiation into classically activated M1‐like microglia, which produce high levels of pro‐inflammatory cytokines and nitric oxide and are thought to contribute to neurological damage in ischemic stroke and Alzheimer's disease. IL‐4 in contrast induces a phenotype associated with anti‐inflammatory effects and tissue repair. We here investigated whether these microglia subsets vary in their K^+^ channel expression by differentiating neonatal mouse microglia into M(LPS) and M(IL‐4) microglia and studying their K^+^ channel expression by whole‐cell patch‐clamp, quantitative PCR and immunohistochemistry. We identified three major types of K^+^ channels based on their biophysical and pharmacological fingerprints: a use‐dependent, outwardly rectifying current sensitive to the K_V_1.3 blockers PAP‐1 and ShK‐186, an inwardly rectifying Ba^2+^‐sensitive K_ir_2.1 current, and a Ca^2+^‐activated, TRAM‐34‐sensitive K_Ca_3.1 current. Both K_V_1.3 and K_Ca_3.1 blockers inhibited pro‐inflammatory cytokine production and iNOS and COX2 expression demonstrating that K_V_1.3 and K_Ca_3.1 play important roles in microglia activation. Following differentiation with LPS or a combination of LPS and IFN‐γ microglia exhibited high K_V_1.3 current densities (∼50 pA/pF at 40 mV) and virtually no K_Ca_3.1 and K_ir_ currents, while microglia differentiated with IL‐4 exhibited large K_ir_2.1 currents (∼ 10 pA/pF at −120 mV). K_Ca_3.1 currents were generally low but moderately increased following stimulation with IFN‐γ or ATP (∼10 pS/pF). This differential K^+^ channel expression pattern suggests that K_V_1.3 and K_Ca_3.1 inhibitors could be used to inhibit detrimental neuroinflammatory microglia functions. GLIA 2016;65:106–121

## Introduction

Microglia are both glia cells and a unique type of mononuclear phagocyte. Recent fate‐mapping studies have shown that microglia are not derived from the bone marrow but originate from haematopoietic stem cells in the yolk sac (Ginhoux et al., 2010) and invade the developing brain after blood vessel formation (Prinz and Priller, 2014; Prinz et al., 2011; Ransohoff and Cardona, 2010). Microglia are long‐lived, able to self‐renew and do not normally seem to be replaced by bone‐marrow derived phagocytes, which only invade the central nervous system under pathological conditions such as stroke. Resting, or more appropriately termed “surveillant” microglia continuously survey their environment with fine cellular processes (Nimmerjahn et al., 2005). Upon detection of signs of injury or inflammation, they retract their ramified processes, round up and transform into “reactive” microglia, which can perform various functions such phagocytosing cellular debris, and producing inflammatory cytokines or neuroprotective factors depending on the stimulus. Similar to macrophages, where the concept of “classically” activated and “alternatively” activated states was first defined (Durafourt et al., 2012; Gordon and Taylor, 2005; Perry et al., 2010), lipopolysaccharide (LPS) and IFN‐γ promote the differentiation of microglia into a cell‐type, which produces high levels of pro‐inflammatory cytokines such as IL‐1β, TNF‐α, IL‐12, IL‐6, and nitric oxide. In contrast, activation with IL‐4 induces a phenotype which is thought to suppress inflammation and promote tissue repair by secreting anti‐inflammatory mediators and neurotrophic factors (Franco and Fernandez‐Suarez, 2015; Kawabori and Yenari, 2014; Perry et al., 2010). In keeping with recent recommendations to abandon the oversimplified M1/M2 terminology for monocyte‐derived macrophages (Murray et al., 2014) also for microglia (Heppner et al., 2015), we are here calling M1‐like microglia M(LPS) or M(IFN‐γ) and M2‐like microglia M(IL‐4) based on the stimulus used to induce polarization (Murray et al., 2014).

Microglia interact with their environment with the aid of a complicated ensemble of receptors, transporters and ion channels which include metabotropic P2Y receptors and ionotropic P2X receptors which detect ATP released from damaged cells, the store‐operated Ca^2+^ channel Orai1, the transient receptor potential (TRP) channels TRPM2, TRPM4 and TRPV2 as well as the K^+^ channels K_ir_2.1, K_V_1.3 and K_Ca_3.1 (Kettenmann et al., 2011; Koizumi et al., 2013; Michaelis et al., 2015). Our laboratory previously described that K^+^ channel expression changes during T and B cell activation and differentiation (Wulff et al., 2003; Wulff et al., 2004). While CCR7^+^ naïve and central memory T cells and IgD^+^ B cells up‐regulate the Ca^2+^‐activated K_Ca_3.1 channel following activation, CCR7^‐^ effector memory T cells and IgD^‐^CD27^+^ memory B cells express high levels of K_V_1.3 following activation and rely on this channel for their Ca^2+^‐signaling events (Beeton et al., 2006; Cahalan and Chandy, 2009; Feske et al., 2015). Based on this differential K^+^ channel expression pattern in T cells, K_Ca_3.1 inhibitors seem to constitute relatively general anti‐inflammatories, while K_V_1.3 channel inhibitors have been proposed for the treatment of T_EM_ cell mediated autoimmune diseases such as multiple sclerosis, rheumatoid arthritis and psoriasis (Chandy et al., 2004). Following efficacy testing in multiple rodent models of these diseases and IND (Investigational New Drug) enabling toxicity studies the K_V_1.3‐selective peptide ShK‐186 (Chi et al., 2012; Tarcha et al., 2012), now called Dalazatide, has recently been found to be effective in phase‐1b studies for psoriasis and is now being developed further for psoriatic arthritis.

We wondered if differential microglia activation would be accompanied by a similar change in K^+^ channel expression. Based on the literature, cultured neonatal mouse or rat microglia are known to increase expression of K_V_1.3 following stimulation with LPS (Norenberg et al., 1994), GM‐CSF (Eder et al., 1995), astrocyte conditioning medium (Schlichter et al., 1996), TGF‐β (Schilling et al., 2000), the HIV‐proteins TAT (Visentin et al., 2001) and glycoprotein 120 (Liu et al., 2012), or after incubation in Teflon bags (Norenberg et al., 1993). Other studies have described K_ir_ currents following stimulation with macrophage colony stimulating factor (M‐CSF) and K_Ca_3.1 currents in lysophosphatidic acid treated mouse microglia (Schilling et al., 2004) or IL‐4 stimulated rat microglia (Ferreira et al., 2014). However, since no study previously directly compared K^+^ channel expression in different polarization states, we differentiated neonatal mouse microglia into M1‐like M(LPS) microglia and M2‐like M(IL‐4) microglia and characterized their K^+^ channel expression by electrophysiology, immunohistochemistry and quantitative PCR. Microglia stimulated with LPS exhibited high K_V_1.3 current densities and virtually no K_Ca_3.1 and K_ir_ currents, while IL‐4 stimulated microglia exhibited K_ir_2.1 currents and down‐regulated K_V_1.3 and K_Ca_3.1 expression. K_Ca_3.1 currents were generally low but moderately increased following stimulation with IFN‐γ alone or ATP. This differential K^+^ channel expression pattern suggests that K_V_1.3 and K_Ca_3.1 inhibitors could be useful to preferentially target detrimental pro‐inflammatory microglia functions in ischemic stroke and other neurological disorders associated with neuroinflammation such as Alzheimer's and Parkinson's disease.

## Materials and Methods

### Primary Cultures of Mouse Microglia

Primary microglia cultures derived from newborn C57BL/6J mice were prepared from mixed glia cultures with the “shaking off” method as described (Maezawa et al., 2011). Floating microglia were harvested between 7 and 14 days in culture and plated at 100,000 – 300,000 cells per well in 24‐well plates in Dulbecco's modified Eagle's medium (DMEM, 25 mM glucose) supplemented with 10% fetal bovine serum (FBS), 1 mM Na^+^ pyruvate, 100 units/ml penicillin, and 100 μg/ml streptomycin. Reseeded cultures were usually ≥ 99% pure based on anti‐Iba1 staining. Cells were differentiated into either M1 or M2 phenotypes by 48 h of stimulation with 300 ng/mL LPS or LPS + 200 ng/mL IFN‐γ (M1) and 20 ng/mL IL‐4 (M2), respectively (Bertrand and Venero, 2013; Xie et al., 2014). Recombinant mouse IFN‐γ and IL‐4 were purchased from Sigma‐Aldrich. In other experiments microglia were stimulated with 500 μM Na_2_ATP or with a combination of 10 nM PMA and 175 nM ionomycin (all Sigma‐Aldrich).

### Human Fetal Microglia

Frozen human microglia of fetal origin were purchased from ZenBio Inc. (Research Triangle Park, NC) and contained 50‐100,000 cells per vial. Freshly thawed microglia were washed once in DMEM and spun down before being used directly for electrophysiology or put in culture. Cells were cultured at 100,000 cells per well overnight (DMEM with 5% FBS) after which either LPS (300 ng/mL) or human IL‐4 (50 ng/mL; Sigma‐Aldrich) were added and cells cultured for an additional 24 h before whole‐cell recording.

### Patch‐Clamp Experiments

Microglia “floating off” from their feeding astrocyte layer or differentiated in 24‐well plates for 40 h and then detached by trypsinization, were washed, attached to poly‐L‐lysine coated glass cover‐slips, and then studied within 20 to 90 min after plating in the whole‐cell mode of the patch‐clamp technique with an EPC‐10 HEKA amplifier. Patch pipettes were pulled from soda lime glass (micro‐hematocrit tubes, Kimble Chase, Rochester, NY) to resistances of 2‐3 MΩ when submerged in the bath solution. These relatively large pipettes were used to assure good access and efficient and complete cell dialysis for internals with high free Ca^2+^ concentrations which show a strong tendency to “reseal” when smaller pipettes are used. The pipette solution contained 145 mM K^+^ aspartate, 2 mM MgCl_2,_ 10 mM HEPES, 10 mM K_2_EGTA and 8.5 mM CaCl_2_ (1 μM free Ca^2+^)_,_ pH 7.2, 290 mOsm. To reduce chloride “leak” currents, we used a Na^+^ aspartate external solution containing 160 mM Na^+^ aspartate, 4.5 mM KCl, 2 CaCl_2_, 1 mM MgCl_2_, 5 mM HEPES, pH 7.4, 300 mOsm. K^+^ currents were elicited with voltage ramps from −120 to 40 mV of 200‐ms duration applied every 10 s. Whole‐cell K_Ca_3.1 conductances were calculated from the slope of the TRAM‐34 sensitive K_Ca_ current between −80 mV and −75 mV where K_Ca_3.1 currents are not “contaminated” by K_V_1.3 (which activates at voltages above −40 mV) or inward‐rectifier K^+^ currents (which activate a voltages more negative than −80 mV). Inward rectifier (K_ir_) currents were measured as Ba^2+^‐sensitive inward currents at −120 mV and K_V_1.3 currents were measured as TRAM‐34‐insensitive, use‐dependent outward currents at +40 mV from the same voltage ramp protocol. In some experiments K_V_ currents were recorded with a KF‐based Ca^2+^‐free internal solution and elicited by voltage steps from −80 to +40 mV as previously described (Wulff et al., 2003). Cell capacitance, a direct measurement of cell surface area, and access resistance were continuously monitored during recordings. K_Ca_3.1 current density was determined by dividing the TRAM‐34‐sensitive slope conductance by the cell capacitance. [We observed that both K_Ca_3.1 and the K_V_1.3 current density decreased with prolonged culture and therefore only used 7‐14 day old cultures for the electrophysiological experiments.].

The K_Ca_3.1 blocker TRAM‐34, and the Kv1.3 blockers PAP‐1 and ShK‐186 were synthesized as previously described (Schmitz et al., 2005; Tarcha et al., 2012; Wulff et al., 2000). The K_ir_ inhibitors BaCl_2_ and ML133 hydrochloride were purchased from Sigma‐Aldrich. Pairwise Student's t‐test was used to determine statistical significance and *p*‐values ≤ 0.05 are considered significant.

### Microglia Activation Assays

Microglia were shaken off their co‐culture layer, and plated at 300,000 cells per well in 6‐well plates or 100,000 cells per well in 24‐well plates in DMEM with 10% FBS. Culture medium was changed 4 h later to fresh DMEM with drugs (15 μM minocycline, 1 μM TRAM‐34, 2 μM PAP‐1 and 10 nM ShK‐186) and incubated for 1 h before LPS (100 ng/ml) or IL‐4 (20 ng/mL) was added. Based on Trypan Blue exclusion these drug concentrations did not affect cell viability over 48 h (data not shown). *Cytokine ELISA* assays were performed in 24‐well plates in DMEM with 5% FBS. Supernatants were collected at 24 and 48 h after stimulation and either used immediately for cytokine assays or stored at −80C° pending analysis. Mouse IL‐1β, IL‐10, TNF‐α and IFN‐γ were assayed using ELISA kits purchased from R&D Systems (Minneapolis, MN) according to the instructions provided by the manufacturer. IFN‐γ and IL‐4 production was below detection. For determining *Nitric oxide* (NO) production supernatant was collected from microglia cultures (1 ×10^5^ cells/24‐well) in Opti‐MEM at 24 h and 48 h and analyzed immediately using the Nitric Oxide Colorimetric Assay Kit (BioVision, Milpitas, CA) according to manufacturer's protocol. NO concentrations were normalized to the amount of total protein determined with a bicinchoninic acid (BCA) based colorimetric protein quantitation kit (ThermoFisher Pierce™ BCA Protein Assay). Briefly, the supernatant was removed and the cells lysed using the Western blot lysis buffer described below. Statistics for cytokine and NO production were performed using One way‐ANOVA (Student‐Newman‐Keuls Method; Sigma Plot software).

For Western blot analysis cells were washed with ice‐cold PBS and incubated with a lysis buffer (150 mM NaCl, 10 mM NaH_2_PO_4_, 1 mM EDTA, 1% TritonX100, 0.5% SDS) with protease inhibitor cocktail and phosphatase inhibitor (Sigma‐Aldrich). Equivalent amounts of protein were analyzed by 4‐15% Tris‐HCl gel electrophoresis (Bio‐Rad, Hercules, CA). Proteins were transferred to polyvinylidene difluoride membranes and probed with antibodies. Visualization was performed using enhanced chemiluminescence (ECL, GE Healthcare Pharmacia). The following primary antibodies were used: anti‐iNOS (1:700), anti‐COX2 (1:1,000, cell signaling), anti‐GAPDH (1:2000); all from Cell Signaling Technology, Danvers, MA). Secondary antibodies were HRP‐conjugated anti‐rabbit or anti‐mouse antibodies (1:1,000, GE Healthcare, Pittsburgh, PA). The Western blot band density for iNOS and COX2 was measured using Image J and normalized to GAPDH. Quantitative PCR experiments for IL‐1β, TNF‐α and iNOS were performed as described below.

### Quantitative PCR

Microglia were plated at 300,000 cells per well in 6‐well plates in DMEM containing 10% FBS and LPS (300 ng/ml) or IL‐4 (20 ng/ml) were added 3 h later. At 0 h, 4 h, 20 h and 40 h after stimulation cells were rinsed several times with PBS, and then lysed and scrapped off using the RTL Plus buffer of the RNeasy Plus Mini kit (Qiagen). RNA was extracted and cDNA was synthesized from 2 μg of total RNA using the iScript Reverse Transcription Supermix (Bio‐Rad). Quantitative PCR (qPCR) was performed using the SsoFast EvaGreen Supermix (Bio‐Rad) in the CFX96 Touch Real‐Time PCR Detection System (Bio‐Rad). The result was normalized to β‐actin. RNA extracted from 14‐day old cortical neuronal cultures prepared from newborn C57BL/6J mice was used as a positive control for the K^+^ channel primers.

The following forward/reverse primer pairs were used.

CD86 (*cd86*): 5′‐CAAGAAGCCGAATCAGCCTA‐3′/5′‐TGGGGTTCAAGTTCCTTCAG‐3′

TNF‐α (*tnfα*): 5′‐GACGTGGAACTGGCAGAAGAG‐3′/5′‐TGCCACAAGCAGGAATGAGA‐3′

IL‐1β (*il1β*): 5′‐CCCCAAGCAATACCCAAAGA‐3′/5′‐TACCAGTTGGGGAACTCTG‐3′

IL‐6 (*il6*): 5′‐GTTCTCTGGGAAATCGTGGA‐3′/5′‐TTCTGCAAGTGCATCATCGT‐3′iNOS (*inos*, *nos2*): 5′‐CGGATAGGCAGAGATTGGAG‐3′/5′‐GTGGGGTTGTTGCTGAACTT‐3′

CD206 (*cd206*): 5′‐TCATCCCTGTCTCTGTTCAGC‐3′/5′‐ATGGCACTTAGAGCGTCCAC‐3′

Arg1 (*arg1*): 5′‐CCAACTCTTGGGAAGACAGC‐3′/5′‐TATGGTTACCCTCCCGTTGA‐3′

YM1 (*ym1*, *chil3*): 5′‐AGGAAGCCCTCCTAAGGACA‐3′/5′‐TGAGTAGCAGCCTTGGAATG‐3′

IGF‐1 (*igf1*): 5′‐TGGATGCTCTTCAGTTCGTG‐3′/5′‐CACAATGCCTGTCTGAGGTG‐3′

K_V_1.3 (*kcna3*): 5′‐ATCTTCAAGCTCTCCCGACCA‐3′/5′‐CGAATCACCATATACTCCGAC‐3′

K_V_1.1 (*kcna1*): 5′‐GAGAATGCGGACGAGGCTTC‐3′/5′‐CCGGAGATGTTGATTACTACGC‐3′

K_V_1.2 (*kcna2*): 5′‐GGTTGAGGCGACCTGTGAAC‐3′/5′‐TCTCCTAGCTCATAAAACCGGA‐3′

K_V_3.1 (*kcnc1*): 5′‐TCGAGGACCCCTACTCATCC‐3′/5′‐CGATTTCGGTCTTGTTCACG‐3′

K_Ca_2.3 (*kcnn3*): 5′‐CCCATCCCTGGAGAGTACAA‐3′/5′‐TTGCTATGGAGCAGCATGAC‐3′

For β‐actin, K_V_1.5, K_Ca_3.1, and K_ir_2.1 we used the commercially available primer set Mouse ACTB (Actin, Beta), Endogenous Control FAM Dye/MGB Probe, Non‐Primer Limited (Invitrogen), PrimePCR SYBR Green Assays. *kcna5* (Assay ID: qMmuCEP0058877), *kcnn4* (Assay ID: qMmuCID0016996), *kcnj2* (Assay ID: qMmuCID0008540) (all Bio‐Rad).

Statistical analysis of qPCR – For each marker a two‐tailed 1‐sample t‐test was performed on the log‐transformed fold‐change value, which amounts to doing a paired test comparing the log‐transformed (unnormalized) values at a given time‐point to the log‐transformed normalization value for that marker for that replication.

### Immunofluorescence (IF) Staining

K_V_1.3 was stained for with a mouse monoclonal anti‐human K_V_1.3 antibody (1:500, AbD Serotec), K_ir_2.1 with a rabbit polyclonal K_ir_2.1 antibody (1:200, AbCam), iNOS with a rabbit polyclonal antibody (1:500, AbCam) and Arginase I with a mouse monoclonal anti‐human Arginase I antibody (1:500, BD Biosciences). Bound primary antibodies were detected by Alexa Fluor^®^546‐conjugated or Alexa Fluor^®^647‐conjugated secondary antibodies (1:500, Life Technologies). Slides were mounted in Fluoromount‐G (Southern Biotech) with DAPI and imaged with a Zeiss LSM‐510 confocal microscope.

## Results

### Primary Microglia Express Small K^+^ Currents

**Figure 1:**
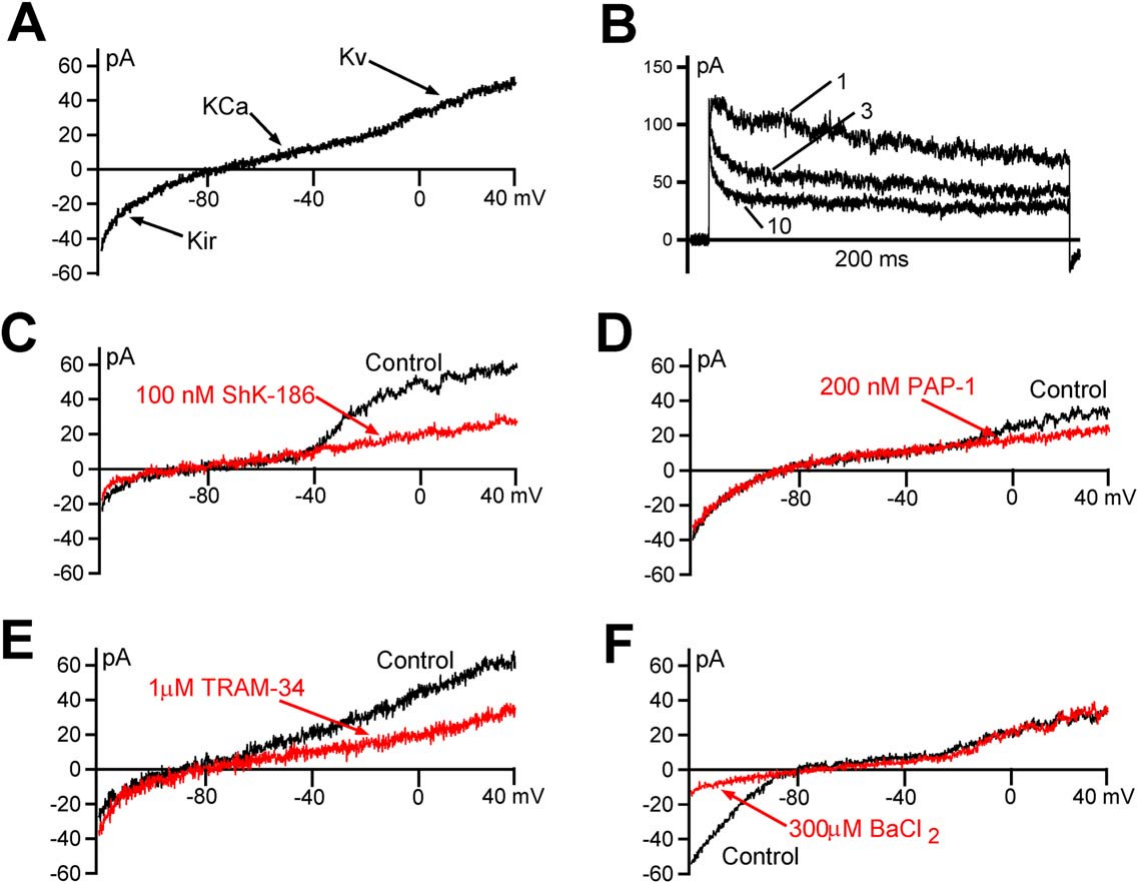
***K^+^*** currents in freshly seeded, unstimulated neonatal mouse microglia. **A**: A typical whole‐cell current consisting of a *K*_ir_, *K*_Ca,_ and K_V_ channel component is visible when a ramp pulse from −120 to +40 mV is applied. **B**: The K_V_ current exhibits the use‐dependence characteristic of K_V_1.3 when 200 ms step pulses from −80 to +40 mV are applied every 1 s (1 = pulse 1; 10 = pulse 10). **C**: The peptidic K_V_1.3 inhibitor ShK‐186 blocks the K_V_ current component of a current elicited by a ramp pulse from −120 to +40 mV. **D**: The small molecule K_V_1.3 inhibitor PAP‐1 blocks the K_V_ current component. **E**: The *K*_Ca_3.1 inhibitor TRAM‐34 blocks the *K*_Ca_ component. **F**: The *K*_ir_ component is blocked by BaCl_2_. [Color figure can be viewed at wileyonlinelibrary.com]

We started by characterizing the basal K^+^ channel expression in primary microglia “floating” out of the astrocyte layer in a mixed glia culture prepared from newborn mice. Cells were plated on poly‐L‐lysine coated glass coverslips and studied by whole‐cell patch‐clamp within 20 min to 90 min to avoid any changes in channel expression through subsequent culture. We decided not to subculture the cells for any prolonged time since the medium composition, especially the amount of serum, and the culture itself had previously been shown to induce partial activation and changes in K^+^ channel expression (Beck et al., 2008). Immediately after plating, microglia were mostly round but quickly flattened out to cells with small ramifications. In this “unstimulated” state three types of small K^+^ currents were typically visible if microglia were dialyzed with 1 μM of free Ca^2+^ through the patch‐pipette and subjected to voltage‐ramps from −120 to +40 mV (Fig. 1A): an inwardly‐rectifying current (K_ir_), a small voltage‐gated current (K_V_), and a voltage‐independent calcium‐activated current component (K_Ca_). The K_V_ current exhibited use‐dependence, a characteristic of K_V_1.3, in which rapid repetitive depolarizing pulses cause a progressive decrease in the current amplitude due to channel trapping in the inactivated state (Fig. 1B). The Kv current was also nearly completely blocked by the Kv1.3 selective peptide‐inhibitor ShK‐186 and the small molecule PAP‐1 (Fig. 1C,D), again suggesting that the K_V_ current is predominantly carried by K_V_1.3. The voltage‐independent calcium‐activated current visible between −80 and −40 mV in Fig. 1E was partially carried by K_Ca_3.1 based on its sensitivity to the K_Ca_3.1 blocker TRAM‐34 and its insensitivity to the K_Ca_2 channel inhibitor apamin (data not shown). The other components of this current were not further identified in this study but it is likely that the calcium‐activated TRPM4 channel (Beck et al., 2008) contributes to the current remaining after application of TRAM‐34 (Fig. 1E). Lastly, the inward‐rectifying K_ir_ current was blocked by Ba^2+^ (Fig. 1F) and ML133 (data not shown) and identified as K_ir_2.1 by qPCR (Fig. 5C).

### K_V_1.3 and K_Ca_3.1 Blockers Inhibit Pro‐Inflammatory Cytokine Production and iNOS and COX2 Expression

**Figure 2:**
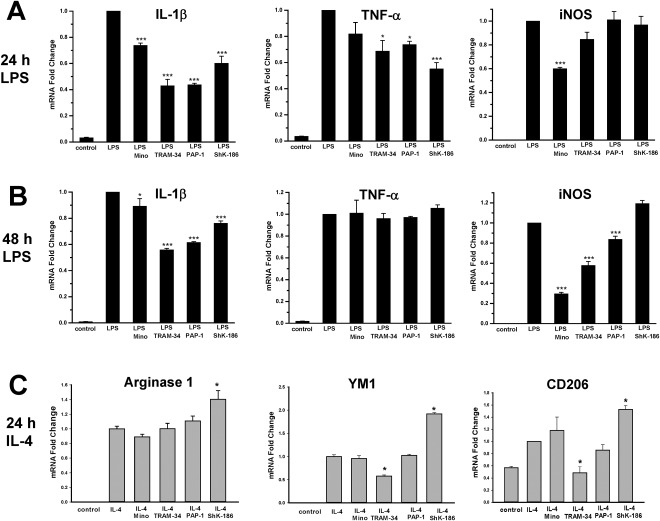
Effect of *K*_V_1.3 and *K*_Ca_3.1 blockers on LPS and IL‐4 stimulated mRNA expression of microglial activation markers. Effect of minocycline (15 μM), TRAM‐34 (1 μM), PAP‐1 (2 μM), and ShK‐186 (10 nM) on LPS stimulated *il1β*, *tnfα*, and *inos* expression at 24 h (**A**) and 48 h (**B**) or on IL‐4 stimulated *arg1*, *cd206*, *ym1* (*chil3)* expression at 24 h (**C**) (*n* = 3). Shown are mean ± S.E.M. ∗*P* < 0.05, ∗∗*P* < 0.01, ∗∗∗*P* < 0.001.

**Figure 3:**
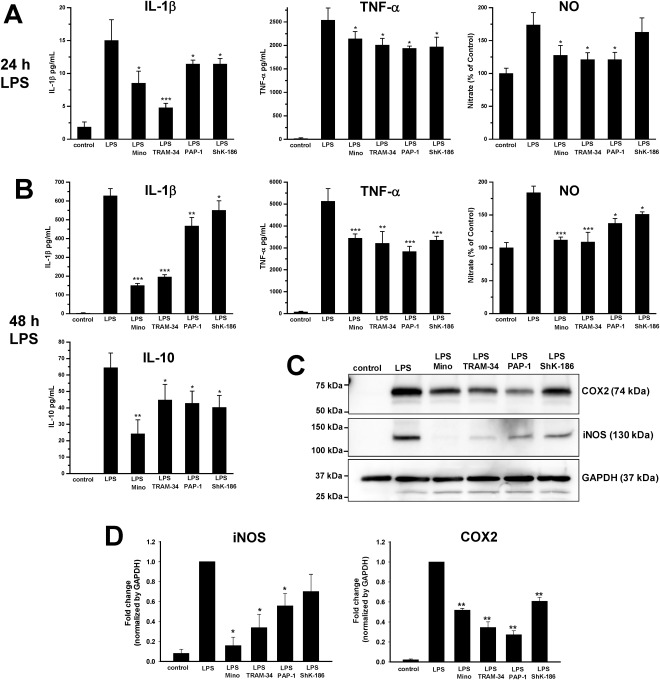
K_V_1.3 and K_Ca_3.1 blockers inhibit LPS stimulated cytokine secretion and iNOS and COX2 expression. Effect of minocycline (15 μM), TRAM‐34 (1 μM), PAP‐1 (2 μM) and ShK‐186 (10 nM) on IL‐1β (*n* = 4), TNF‐α (*n* = 4), and NO production (*n* = 4 or 6) at 24 h (**A**) and 48 h (**B**) after stimulation with LPS (100 ng/mL). Representative Western blot (**C**) and quantification of Western blot analysis (**D**) for COX2 and iNOS of lysates from microglia exposed to LPS for 48 h. Shown are mean ± S.D. (*n* = 3). ∗*P* < 0.05, ∗∗*P* < 0.01, ∗∗∗*P* < 0.001.

In order to confirm the previously reported importance of K^+^ channels for pro‐inflammatory microglia functions (Fordyce et al., 2005; Khanna et al., 2001) we stimulated microglia for 24 h and 48 h with the gram‐negative cell wall component lipopolysaccharide (LPS) in the presence and absence of K_V_1.3 blockers (PAP‐1 and ShK‐186), K_Ca_3.1 blockers (TRAM‐34), K_ir_2.1 blockers (Ba^2+^ and ML133) and the widely used microglia inhibitor minocycline (Möller et al., 2016). Unfortunately, BaCl_2_ (250 μM and 1 mM) and ML133 (10 and 25 μM) affected microglia viability over 48 h making it impossible for us to evaluate the effect of pharmacological K_ir_2.1 inhibition. Similar to minocycline, K_Ca_3.1 and K_V_1.3 blockers reduced *IL‐1β* and *TNF‐α* expression as determined by qPCR (Fig. 2A) as well as IL‐1β and TNF‐α secretion as determined by ELISA measured 24 h after LPS stimulation (Fig. 3A). At the later time point (48 h), both blockers still significantly reduced IL‐1β and TNF‐α secretion (Fig. 3B) but no longer affected TNF‐α messenger RNA levels (Fig. 2B). LPS stimulation also induced low levels of IL‐10 secretion (∼80 pg/mL compared to ∼600 pg/mL IL‐1β or ∼5 ng/mL TNF‐α) at 48 h which was most strongly suppressed by minocycline but also reduced by K^+^ channel inhibition (Fig. 3B). Both K_V_1.3 and K_Ca_3.1 inhibitors also reduced *iNOS* expression at the mRNA level (Fig. 2) and NO production at 24 and 48 h (Fig. 3) following LPS stimulation. Western blotting at 48 h (Fig. 3C and 3D) further revealed a strong reduction in iNOS protein expression by minocycline and TRAM‐34 and reduced COX‐2 expression, especially with PAP‐1 (Fig. 3C,D).

Interestingly, K_V_1.3 and K_Ca_3.1 inhibitors exhibited a somewhat differential effect on IL‐4 induced activation markers (Fig. 2C). Similar to minocycline, the K_V_1.3 inhibitors PAP‐1 and ShK‐186 did not affect or increased *arginase‐1*, *YM‐1* and *CD206* mRNA expression, while the K_Ca_3.1 blocker TRAM‐34 reduced *YM1* and *CD206* mRNA expression at 24 h (Fig. 2C). The later findings corroborate a recent report that K_Ca_3.1 inhibition with TRAM‐34 can switch the phenotype of glioma infiltrating microglia/macrophages away from a tumor‐promoting to a more pro‐inflammatory anti‐tumor phenotype (Grimaldi et al., 2016).

### M(LPS) Microglia Exhibit Large K_V_1.3 Currents, While M(IL‐4) Microglia Exhibit Large K_ir_2.1 Currents

**Figure 4:**
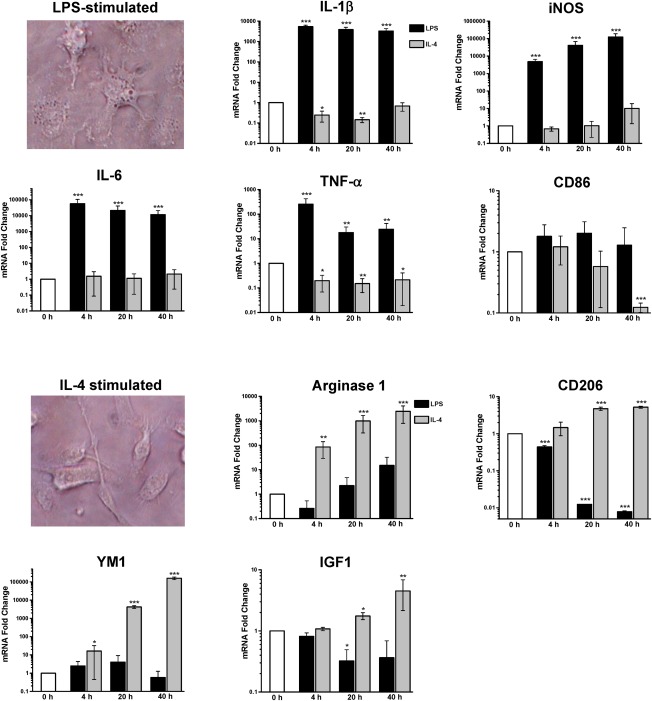
M1 and M2 marker expression in neonatal mouse microglia following stimulation with LPS or IL‐4**.** Both stimuli induced the expected changes in cell shape (“fried egg” shape with LPS and spindly shaped with IL‐4) and differentially affected expression of the M1‐related genes *il1β*, *il6*, *tnfα*, *inos*, and *cd86* and the M2‐related genes *arg1*, *cd206*, *ym1* (*chil3)*, and *igf1* at 4, 20, and 40 h after stimulation (300 ng/mL LSP; 20 ng/mL IL‐4). Shown are mean ± S.E.M. (*n* = 3). ∗*P* < 0.05, ∗∗*P* < 0.01, ∗∗∗*P* < 0.001. [Color figure can be viewed at wileyonlinelibrary.com]

**Figure 5:**
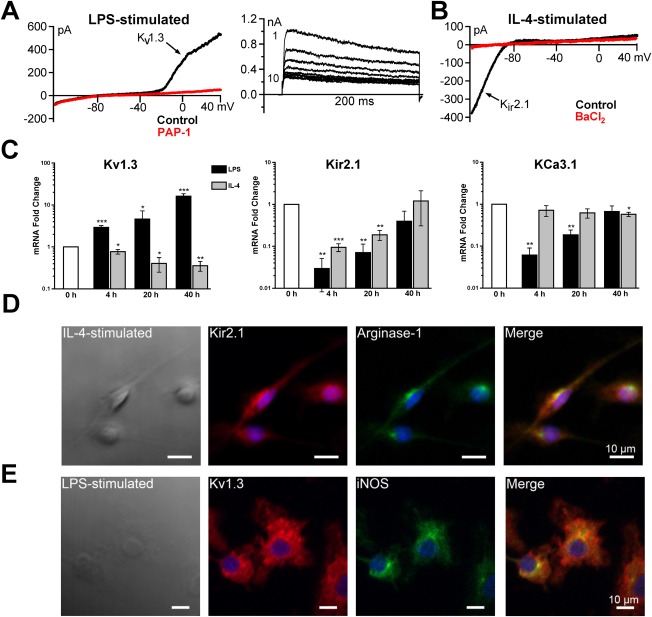
LPS and IL‐4 stimulated microglia exhibit a differential K^+^ channel expression profile. **A**: LPS‐stimulated microglia exhibit large PAP‐1 sensitive and use‐dependent K_V_1.3 currents 40–48 h after stimulation (recording condition of ramp and step pulses as described in Fig. 1; 200 nM PAP‐1). **B**: IL‐4 stimulated microglia exhibit large Ba^2+^ sensitive *K*_ir_2.1 currents 40–48 h after stimulation. **C**: K_V_1.3 (*kcna3*), *K*_ir_2.1 (*kcnj2*), and *K*_Ca_3.1 (*kcnn4*) mRNA levels at 4, 20, and 40 h after stimulation with LPS or IL‐4. **D**: Immunofluorescence showing *K*_ir_2.1 staining on arginase‐1 positive M(IL‐4) microglia. **E**: Immunofluorescence showing *K*_V_1.3 staining on iNOS positive M(LPS) microglia. Shown are mean ± S.E.M. (*n* = 3). ∗*p* < 0.05, ∗∗*p* < 0.01, ∗∗∗*p* < 0.001. [Color figure can be viewed at wileyonlinelibrary.com]

We next induced polarization in microglia by treating cells either with LPS or with the immunomodulatory cytokine IL‐4. Both stimuli induced the expected changes in cell shape and gene expression. LPS‐stimulation induced the characteristic “fried egg” shape and increased expression of the M1‐related genes *il1β*, *il6*, *tnfα*, and *inos (nos2)* (Fig. 4). In contrast, IL‐4 induced cells were more ramified and spindly shaped and showed reduced expression of the M1‐gene *cd86* and increased expression of the M2‐genes *arg1*, *cd206*, *ym1* (*chil3*), and *igf1* (Fig. 4). This polarization was accompanied by a striking change in functional K^+^ channel expression. M(LPS) microglia expressed large K_V_1.3 currents 40 h after stimulation, which were sensitive to PAP‐1 (Fig. 5A) and ShK‐186 (not shown) and highly use‐dependent (Fig. 5A), but virtually no K_Ca_3.1 and K_ir_ currents. In contrast, M(IL‐4) microglia typically showed Ba^2+^ sensitive K_ir_ currents and virtually no K_V_1.3 currents (Fig. 5B). Electrophysiology was performed between 40 and 48 h because we wanted to be sure that microglia were fully differentiated. [Please note that message for some of the M2‐markers such as YM1, arginase‐1 and IGF1 still increased 5 to 10‐fold between 20 and 40 h (Fig. 4).]

The differences in functional K^+^ channel expression measured by electrophysiology were also observed at the mRNA level by qPCR. Following LPS stimulation K_V_1.3 mRNA levels increased dramatically (∼40‐fold at 40 h), while K_Ca_3.1 and K_ir_2.1 mRNA levels decreased at 4 and 20 h and then returned to baseline at 40 h (Fig. 5C). IL‐4 stimulation in contrast induced much smaller and less rapid changes in K^+^ channel mRNA. Messenger RNA levels of K_V_1.3 decreased compared to resting microglia, while K_ir_2.1 mRNA levels first decreased and then increased 2‐fold at 40 h (Fig. 5C). K_Ca_3.1 levels remained unaffected by IL‐4 stimulation (Fig. 5C). Immunofluorescence staining performed 48 h after IL‐4 stimulation showed K_ir_2.1 staining on arginase‐1 positive, spindly shaped cells (Fig. 5D), while LPS stimulated cells exhibited strong staining for both K_V_1.3 and iNOS (Fig. 5E).

**Figure 6:**
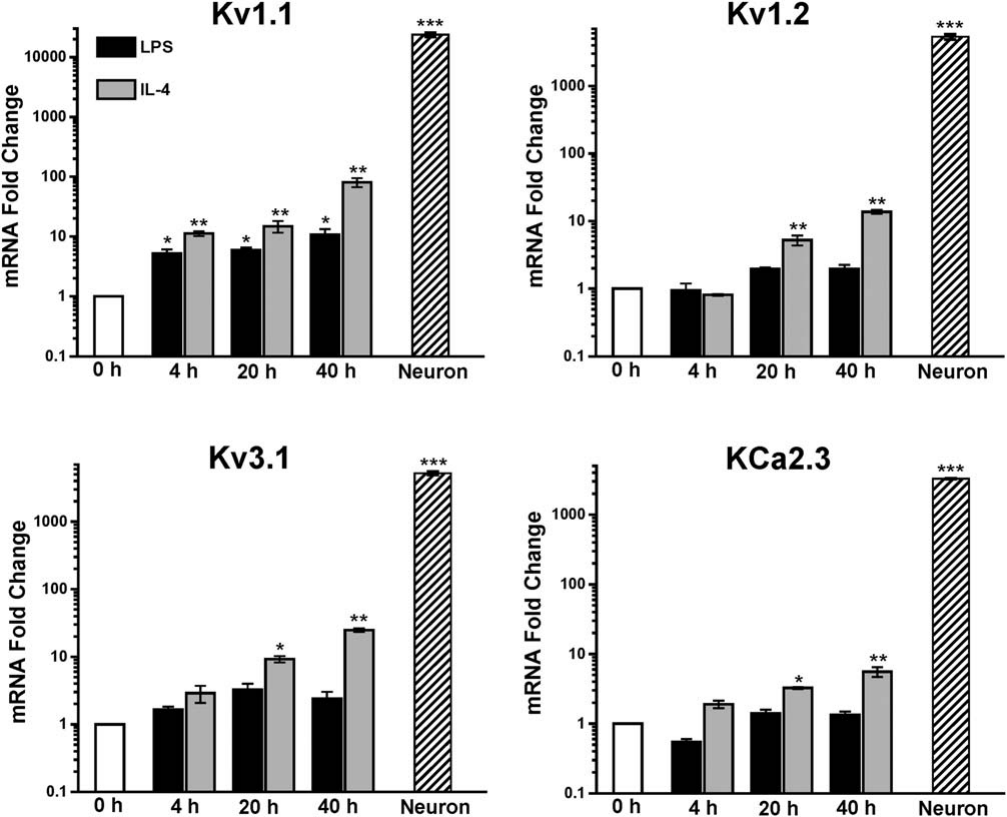
mRNA expression of other *K* ^+^ channels. *K*_V_1.1 (*kcna1*), *K*_V_1.2 (*kcna2*), *K*_V_3.1 (*kcnc1*), and *K*_Ca_2.3 (*kcnn3*) mRNA levels at 4, 20, and 40 h after stimulation with LPS or IL‐4. RNA from cortical neuron cultures was used as a positive control. *K*_V_1.5 (*kcna5*) mRNA was not detectable in microglia but present in neurons. Shown are mean ± S.E.M. (*n* = 3). ∗*P* < 0.05, ∗∗*P* < 0.01, ∗∗∗*P* < 0.001.

A broader analysis of microglial K^+^ channel expression using RNA extracted from 14‐day old cortical neuron cultures as a positive control revealed low, but detectable levels of K_V_1.1, K_V_1.2, K_V_3.1 and K_Ca_2.3 mRNA (Fig. 6). However, message levels for these channels only moderately increased at 20 or 40 h after IL‐4 stimulation and did not change following LPS treatment. Interestingly, K_V_1.5, which was strongly expressed in cortical neurons was not detectable in our hands with a commercial primer (data not shown, see Methods for primer sequence). However, compared to cortical neurons (Fig. 6) even the highest RNA levels following IL‐4 stimulation of these channels are 200‐300 fold lower suggesting very low, if any, functional expression as proteins.

**Figure 7:**
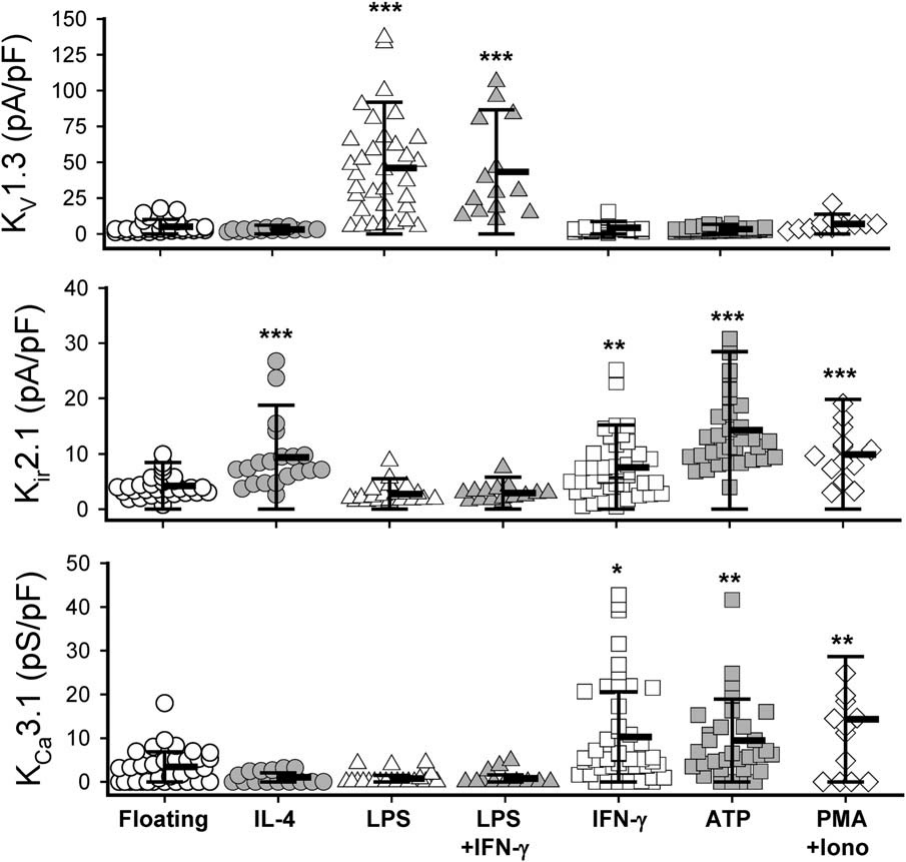
Summary of functional *K*_V_1.3, *K*_Ca_3.1, and *K*_ir_2.1 expression levels measured by whole‐cell patch‐clamp 40–48 h after stimulation with the respective stimulus**.** Top: *K*_V_1.3 current density at +40 mV for floating microglia (5.0 ± 4.4 pA/pF, *n* = 35) and microglia treated with IL‐4 (3.0 ± 1.0 pA/pF, *n* = 15, *P* = 9.1 x 10^−2^), LPS (46.0 ± 35.4 pA/pF, *n* = 33, *P* = 4.1 x 10^−9^), LPS + IFN‐γ (43.2 ± 33.6 pA/pF, *n* = 14, *P* = 2.6 x 10^−8^), IFN‐γ (4.2 ± 3.0 pA/pF, *n* = 41, *P* = 3.8 x 10^−1^), MgATP/Na_2_ATP (3.3 ± 1.5 pA/pF, *n* = 32, *P* = 4.1 x 10^−2^) and PMA/ionomycin (6.8 ± 5.0 pA/pF, *n =* 12, *P* = 2.4 x 10^−1^). Middle: *K*_ir_ current density at −120 mV for floating microglia (4.2 ± 2.0 pA/pF, *n =* 30) and microglia treated with IL‐4 (9.4 ± 6.2 pA/pF, *n =* 20, *P* = 1.1 x 10^−4^), LPS (2.8 ± 1.8 pA/pF, *n =* 21, *P* = 9.1 x 10^−3^), LPS + IFN‐γ (2.9 ± 1.5 pA/pF, *n =* 15, *P* = 3.0 x 10^−2^), IFN‐γ (7.6 ± 5.6 pA/pF, *n =* 50, *P* = 2.6 x 10 ^− 3^), MgATP/Na_2_ATP (14.2 ± 7.0 pA/pF, *n =* 33, *P* = 2.2 x 10^−10^) and PMA/ionomycin (9.9 ± 4.8 pA/pF, *n =* 13, *P* = 2.4 x 10^−6^). *Bottom*) K_Ca_3.1 current density determined from the slope of ramp current between −80 mV to −70 mV: for floating microglia (3.4 ± 3.9 pS/pF, *n =* 33) and microglia treated with IL‐4 (1.1 ± 1.3 pS/pF, *n =* 15, *P* = 2.6 x 10 ^− 2^), LPS (0.7 ± 1.5 pS/pF, *n =* 20, *P* = 5.4 x 10 ^− 3^), LPS + IFN‐γ (0.8 ± 1.6 pS/pF, *n =* 14, *P* = 2.0 x 10 ^− 2^), IFN‐γ (10.2 ± 11.5 pS/pF, *n =* 47, *P* = 1.5 x 10^−3^), MgATP/Na_2_ATP (9.4 ± 9.0 pS/pF, *n =* 32, *P* = 9.0 x 10^−4^) and PMA/ionomycin (14.3 ± 17.6 pA/pF, *n =* 13, *P* = 1.4 x 10^−3^). All data are mean ± S.D. ∗*P* < 0.05, ∗∗*P* < 0.01, ∗∗∗*P* < 0.001.

Figure 7 shows a summary of the functional K_V_1.3, K_Ca_3.1, and K_ir_2.1 expression levels measured by electrophysiology between 40 and 48 h after stimulation. When current amplitudes are normalized to cell capacitance to correct for differences in cell size and determine channel density, it becomes apparent that IL‐4 stimulation induces a statistically significant increase in K_ir_2.1 current density, while LPS or the combination of LPS and IFN‐γ induces significant increases in K_V_1.3 current density. Interestingly, K_Ca_3.1 current density did not change significantly in comparison to unstimulated microglia following treatment with LPS or IL‐4.

### KCa3.1 Current Density Increases following Stimulation with IFN‐γ or ATP

**Figure 8:**
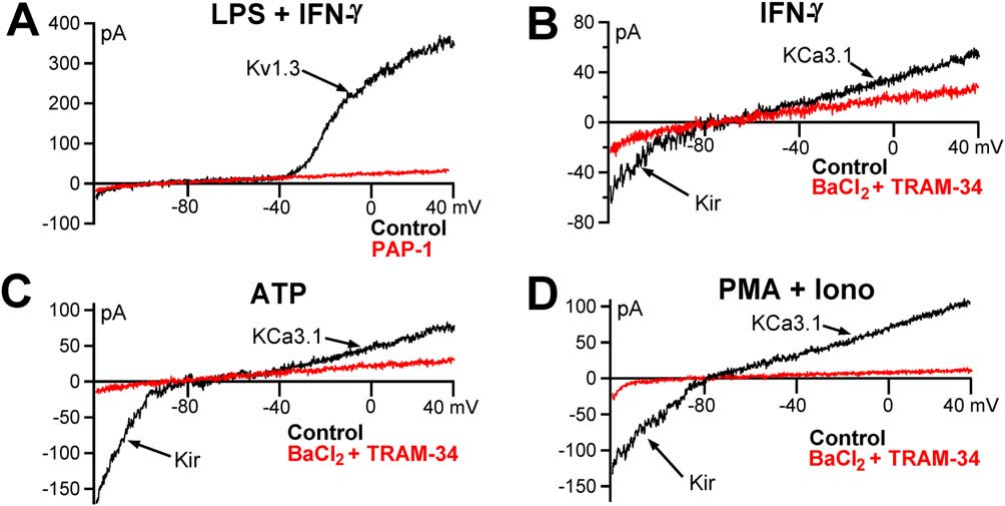
KCa3.1 currents increase following stimulation with IFN‐γ, ATP or PMA and ionomycin. **A**: Stimulation with a combination of LPS and IFN‐γ for 40–48 h induces a large *K*_V_1.3 current. The current can be blocked by 200 nM PAP‐1. **B,C**: Stimulation with IFN‐γ (200 ng/mL) alone or ATP (500 μM) induces a combination of *K*_ir_2.1 and *K*_Ca_3.1. The remaining current after application of 1 μM of TRAM‐34 and 300 μM BaCl_2_ is probably carried by TRPM4. **D**: Stimulation with PMA (10 nM) and ionomycin (175 nM) induces *K*_ir_2.1 and *K*_Ca_3.1 currents. [Color figure can be viewed at wileyonlinelibrary.com]

For comparison we also stimulated microglia with IFN‐γ alone and found that in contrast to the combination of LPS and IFN‐γ, this stimulus did not increase K_V_1.3 current density, but instead induced significant increases in K_Ca_3.1 and K_ir_2.1 current density (see Fig. 7 for statistics and Fig. 8 for representative current recordings). Similar increases in functional K_Ca_3.1 and K_ir_2.1 expression were observed when microglia were stimulated with 500 μM of the P2Y/X receptor agonist ATP or the combination of the PKC activator PMA and the calcium ionophor ionomycin (Figs. 7 and 8). Taken together these results demonstrate that microglial K^+^ channel expression is highly stimulus dependent. While “classically” activated M(LPS) microglia exhibit large K_V_1.3 currents and virtually no K_ir_2.1 and K_Ca_3.1 currents, “alternatively” activated M(IL‐4) microglia are dominated by K_ir_2.1. However, activation with other stimuli like the danger signal ATP or the inflammatory cytokine IFN‐γ induces an “intermediate” phenotypes characterized by low K_V_1.3 current densities and moderately high K_Ca_3.1 and K_ir_2.1 current densities.

### Human Fetal Microglia Express Kv1.3

**Figure 9:**
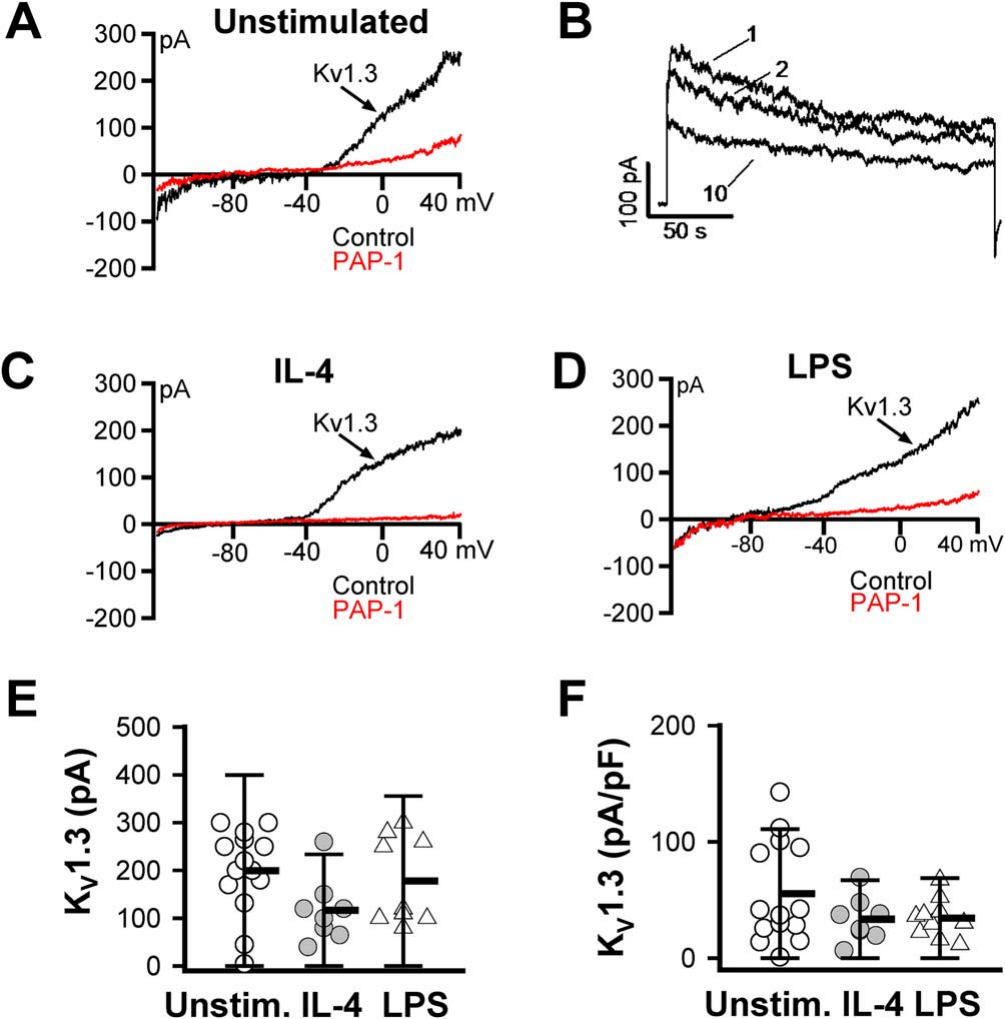
K^+^ currents in human fetal microglia. **A**: Whole‐cell recording from an unstimulated human fetal microglia showing predominantly a Kv1.3 current component when ramp pulse from −120 to +40 mV is applied. This outward current is blocked by 200 nM PAP‐1. **B**: The K_V_ current exhibits the use‐dependence characteristic of K_V_1.3 when 200‐ms step pulses from −80 to +40 mV are applied every 1 s. **C**,**D**: Human microglia retain K_V_1.3 current expression 24 h after treatment with IL‐4 and LPS. **E**, **F**: Summary of functional K_V_1.3 expression levels measured by whole‐cell patch‐clamp. **E**: K_V_1.3 current amplitude at +40 mV for unstimulated microglia (199.8 ± 89.4 pA, *n =* 14), microglia treated with IL‐4 (116.8 ± 67.4 pA, *n =* 8, *P* = 3.4 x 10^−2^) and LPS (177.8 ± 91.4 pA, *n =* 9, *P* = 5.7 x 10^−2^). **F**: K_V_1.3 current density at +40 mV for unstimulated microglia (55.4 ± 43.8 pA/pF, *n =* 14), microglia treated with IL‐4 (33.5 ± 19.4 pA/pF, *n =* 8, *P* = 19.7 x 10^−2^) and LPS (34.4 ± 16.6 pA/pF, *n =* 9, *P* = 16.4 x 10^−2^). [Color figure can be viewed at wileyonlinelibrary.com]

We and others have previously used immunohistochemistry to demonstrate K_V_1.3 and K_Ca_3.1 expression on activated microglia in human ischemic infarcts (Chen et al., 2015) and K_V_1.3 expression on microglia surrounding amyloid‐plaques in Alzheimer's disease (Rangaraju et al., 2015). However, these studies did not investigate any correlation between K^+^ channel and M1/M2 marker expression. In order to test if a similar association of K_V_1.3 with M1‐like M(LPS) and K_ir_2.1 and M2‐like M(IL‐4) exists in human microglia, we obtained fetal human microglia from a commercial source and patch‐clamped the microglia before and after activation with LPS and IL‐4. In contrast to neonatal mouse microglia, which attach and flatten out quickly after plating, many of the human fetal microglia remained floating and only roughly 20% attached overnight. We therefore subjected fetal human microglia to whole‐cell patch‐clamp directly after thawing by attaching them to poly‐L‐lysine coated coverslips. The cells had an average capacitance of 5.3 ± 3.3 pF (*n* = 15) and already displayed K_V_1.3 currents (Fig. 9A,F) that were much more sizable than the K_V_1.3 currents in floating neonatal mouse microglia (Figs. 1 and 7). We further removed “floaters” from overnight cultures and stimulated the remaining cells with LPS and IL‐4. This stimulation did not result in the dramatic morphological changes observed with neonatal mouse microglia and K_V_1.3 current density did not change significantly 24 h after LPS and IL‐4 stimulation (Fig. 9E,F). Cells became apoptotic at 48 h after stimulation suggesting that they might have already been activated by the undisclosed isolation procedure of the vendor or a pathophysiological condition in the fetal source. The limited number of cells and their extreme fragility when using Ca^2+^ containing pipette solutions only allowed us to study K_V_1.3 currents with a KF based pipette solution.

## Discussion

Similar to macrophages, microglia can be polarized into a pro‐inflammatory and predominantly neurotoxic phenotype and an alternatively activated, anti‐inflammatory phenotype that seems to be promoting resolution of tissue damage and repair (Durafourt et al., 2012; Franco and Fernandez‐Suarez, 2015; Gordon and Taylor, 2005; Perry et al., 2010). The data presented here, demonstrate that this polarization also induces a differential K^+^ channel expression pattern. While M(LPS) microglia exhibit high current densities of the voltage‐gated K^+^ channel K_V_1.3, IL‐4 stimulation leads to a moderate increase in functional expression of the inward‐rectifier K_ir_2.1 and a down‐regulation of K_V_1.3 and K_Ca_3.1. This high K_V_1.3 expression in microglia stimulation with the TLR‐4 ligand LPS is reminiscent of the high K_V_1.3 expression previously reported in activated CCR7^‐^ effector memory T cells (Beeton et al., 2006; Wulff et al., 2003) and IgD^‐^CD27^+^ class‐switched memory B cells (Feske et al., 2015; Wulff et al., 2004). In keeping with these previous studies, which showed that K_V_1.3 blockers preferentially affect the proliferation of these T and B cell subsets and inhibit the production of inflammatory Th1 and Th17 cytokines *in vitro* and *in vivo* (Azam et al., 2007; Beeton et al., 2006; Gocke et al., 2012; Koch Hansen et al., 2014), we here found that K_V_1.3 blockers inhibit the production of the pro‐inflammatory cytokines IL‐1β and TNF‐α and of NO in microglia. Interestingly, K_Ca_3.1 inhibition had very similar effects despite the relatively low K_Ca_3.1 current density before and after LPS stimulation suggesting that a small number of K_Ca_3.1 channels can have a profound effect on microglia functions.

The LPS and LPS plus IFN‐γ induced M1‐like state and the IL‐4 induced M(IL‐4) state (Franco and Fernandez‐Suarez, 2015) are of course two extremes of the diverse continuum of microglial activation states that exist *in vivo* where overlapping phenotypes co‐expressing M1 and M2 markers often predominate, especially in human inflammatory and neurodegenerative diseases (Prinz and Priller, 2014; Vogel et al., 2013). When patch‐clamping acutely isolated CD11b^+^ microglia/macrophages from the brains of mice subjected to either ischemic stroke or intraventricular LPS injection, our own group recently observed four types of K^+^ channel expression patterns (Chen et al., 2015). Microglia acutely isolated from non‐infarcted, normal brains exhibited very small K^+^ currents, which on average were even smaller than what we report here for “floating” microglia from neonatal cultures [∼5 pA/pF K_V_1.3, 2 pA/pF K_ir_ and 29 pS/pF K_Ca_3.1]. Ischemic stroke increased functional expression of all three channels studied here and we observed activated microglia predominantly expressing K_ir_2.1, which would correspond to the IL‐4 stimulated M(IL‐4) microglia described here, but also cells predominantly showing large K_V_1.3 currents similar to the M(LPS) cells in this study as well as cells exhibiting various combinations of K_V_1.3, K_Ca_3.1 and K_ir_2.1 currents (Chen et al., 2015). Interestingly, microglia isolated from LPS injected brains typically expressed either large K_V_1.3 currents or a combination of K_V_1.3 and K_Ca_3.1 but virtually no K_ir_ currents. So purely based on their K^+^ channel pattern, extreme M(LPS) and M(IL‐4)‐polarized microglia seem to be present under pathophysiological conditions but are accompanied by microglia exhibiting intermediate phenotypes. We therefore also stimulated cultured neonatal microglia with other stimuli such as the inflammatory cytokine IFN‐γ without LPS, the “danger signal” ATP or a combination of the PKC activator PMA and the calcium ionophor ionomycin. These stimuli induced increased current densities of both K_ir_2.1 and K_Ca_3.1 but not K_V_1.3 (Figs. 7 and 8).

As mentioned in the introduction, K^+^ channel expression in microglia has previously been found to vary widely depending on the culture conditions, the mode of stimulation and the species (Kettenmann et al., 2011), in line with the recently proposed more graded and stimulus‐based nomenclature scheme for macrophages (Murray et al., 2014) and presumably also microglia (Heppner et al., 2015). While our findings largely agree with previous *in vitro* studies on cultured neonatal mouse microglia and our own *in vivo* studies with acutely isolated adult murine microglia (Chen et al., 2015), there are some differences to previously reported results with rat microglia. We here had no indication of significant functional expression of the voltage‐gated K^+^ channel K_V_1.5, which had been observed in freshly tissue printed rat microglia but which then vanished from the cell surface as the microglia were put into culture (Kotecha and Schlichter, 1999). We did occasionally observe a small none‐inactivating K_V_ current in some cells, which could have been carried by K_V_1.5 or K_V_1.3/K_V_1.5 heteromultimers based on previous studies (Vicente et al., 2006). However, given the fact that we were not able to detect any K_V_1.5 message (Fig. 6) we believe it more likely that this current was carried by K_V_1.1, K_V_1.2 or K_V_3.1, all channels for which there are low levels of mRNA detectable. But overall the none‐inactivating component never was more than a minor current component compared to the clearly use‐dependent and ShK‐186 sensitive major K_V_ current component carried by K_V_1.3. We further observed a small, very rapidly inactivating current component in floating microglia (visible in Fig. 1B after 3 and 10 consecutive pulses), which we could not identify and which vanished after activation with LPS (Fig. 5A). We also did not observe any apamin‐sensitive small‐conductance Ca^2+^‐activated K_Ca_2 or SK channels which also have been reported in cultured primary rat microglia (Khanna et al., 2001) or the MSL‐9 rat microglial cell line (Siddiqui et al., 2014) despite the fact that low levels of K_Ca_2.3 message were detectable in our study. With respect to polarizing stimuli, both LPS and IFN‐γ have been reported to increase K_V_1.3 expression in cultured neonatal rat microglia as early as 1992 (Norenberg et al., 1992, 1994), while findings with IL‐4 again seem to differ between mice and rats. IL‐4 stimulation of rat microglia has recently been reported to not change K_ir_2.1 expression at 6 and 24 h after stimulation at both the mRNA and current level (Lam and Schlichter, 2015). However, we here found that 40‐48 h after IL‐4 stimulation K_ir_2.1 current levels were significantly increased (Fig. 7) even if mRNA only showed a trend towards a none‐significant 2‐fold increase 40 h after stimulation (Fig. 5). Our findings are also at odds with another report from the same laboratory, which recently described increased K_Ca_3.1 expression in IL‐4 stimulated cultured neonatal rat microglia (Ferreira et al., 2014). Interestingly, these investigators reported that the K_Ca_3.1 current was not active following dialysis with 1 μM of free Ca^2+^ alone (which could have been caused by insufficient dialysis through their much smaller pipettes) but only became visible in the presence of K_Ca_ channel activators like riluzole or NS309 (Ferreira et al., 2014). In our own hands, the K_Ca_3.1 activators riluzole, SKA‐31 and SKA‐121 (Coleman et al., 2014) or working with internals containing 10 or even 30 μM free Ca^2+^ did not induce K_Ca_3.1 currents in IL‐4 stimulated mouse microglia in keeping with the low level of K_Ca_3.1 mRNA in these cells. Increased K_Ca_3.1 expression in rat microglia was specifically mediated through the type 1 and not the type 2 IL‐4 receptor (Ferreira et al., 2014) and it is of course possible that there are species differences between mice and rats in the importance of the down‐stream signaling and the resulting gene expression of the two IL‐4 receptors in microglia (Ferreira et al., 2014; Gadani et al., 2012). Future studies should therefore directly compare K^+^ channel expression in different species in inflammatory versus M(IL‐4) microglia and also investigate human microglia.

We here started to address this question by patch‐clamping human fetal microglia but can basically only conclude from these experiments that human microglia can express K_V_1.3. It was of course interesting to observe that “floating” fetal human microglia expressed much higher levels of K_V_1.3 than unstimulated neonatal mouse microglia, but at this point it is impossible to conclude whether this K_V_1.3 expression is a consequence of the isolation procedure employed by the commercial vendor or a general characteristic of more proliferative fetal microglia. The difference could also constitute a true species difference between humans and rodents reflecting the previously observed differences between human and rodent T cells. While human T cells already express roughly 250 K_V_1.3 channels in the resting state, rat and mouse T cells typically only express a very small number of channels (∼5) in the resting state and then up‐regulate K_V_1.3 expression after activation (Beeton and Chandy, 2005; Beeton et al., 2001; Decoursey et al., 1987). Interestingly, another group very recently patch‐clamped adult human microglia from neocortical tissue surgically removed from epilepsy patients and found high K_Ca_3.1 current densities (∼580 per cell), which, similar to our observations here, did not significantly change with LPS or IL‐4 treatment (Blomster et al., 2016).

In summary, we here demonstrated that pro‐inflammatory M(LPS) and M(LPS + IFN‐γ) microglia express high levels of the voltage‐gated K^+^ channel K_V_1.3, while the 3^rd^ type of inflammatory microglia, IFN‐γ‐stimulated M(IFN‐γ) microglia express a combination of K_Ca_3.1 and K_ir_2.1 similar to ATP‐stimulated microglia (Fig. 7). In keeping with this expression pattern both K_V_1.3 and K_Ca_3.1 inhibitors suppressed pro‐inflammatory cytokine and NO production as effectively as the widely used microglia inhibitor minocycline.

Since both K_V_1.3 (Beeton et al., 2006; Pereira et al., 2007) and K_Ca_3.1 (Ataga et al., 2008; Maezawa et al., 2012) blockers have been shown to be relatively safe and well tolerated *in vivo* we would like to suggest K_V_1.3 and K_Ca_3.1 inhibition as pharmacological approaches to preferentially inhibit detrimental microglia responses in stroke and other brain disorders associated with neuroinflammation (Dale et al., 2016). Supporting our proposal of K_V_1.3 inhibitors for reducing detrimental microglia functions are findings from our own group that PAP‐1 reduces infarct areas and improves neurological deficit in ischemic stroke in rats (Chen et al., 2013) as well as a report from Peng et al. that ShK‐170 (a close derivative of ShK‐186) protects mice from microglia mediated radiation‐induced brain injury (Peng et al., 2014). Similarly, K_Ca_3.1 blockers have been demonstrated to reduce microglia activation and cytokine production in MOG‐induced experimental autoimmune encephalomyelitis (EAE) (Reich et al., 2005), prevent microglia activation and retinal ganglion cell degeneration after optic nerve transection (Kaushal et al., 2007), and to reduce infarct area in models of traumatic brain injury (Mauler et al., 2004) or stroke (Chen et al., 2011). Moreover, our own group recently demonstrated that both genetic K_Ca_3.1 deletion and pharmacological K_Ca_3.1 blockade with TRAM‐34 started 12 h after reperfusion reduced inflammatory brain cytokine production and microglia activation, and improved neurological deficit in a mouse model of ischemic stroke (Chen et al., 2015). Whether one type of K^+^ channel blocker (K_V_1.3 or K_Ca_3.1) is superior to the other type or offers any advantages over minocycline *in vivo* will have to be investigated in future.
